# Supplementary material for: A Likelihood Approach for Real-Time Calibration of Stochastic Compartmental Epidemic Models
Source: PLoS Comput Biol. 2017 Jan 17;13(1):e1005257. doi: 10.1371/journal.pcbi.1005257 (PMC5240920; doi:10.1371/journal.pcbi.1005257)
Supplement: S1 File — (TAR.GZ) [file pcbi.1005257.s014.tar.gz › HSPH_Online-SI-Revision/EnKF07/n100-mild/n100-mild_EnKF2_table4.pdf]

1	newly 3 weeks spec	734.	1879.22	{116., 4776.}	186.744	0.410562	newly 3 weeks cumul	3465.	12 311.8	{418., 16 380.}	286.379	0.477362	newly 5 weeks spec	287.	589.72	{58., 1019.}	144.237	0.419667	newly 5 weeks cumul	4194.	13 914.3	{630., 22 232.}	259.425	0.452071	newly 48 weeks cumul	4721.	15 220.7	{1495., 27 398.}	244.965	0.42505
2	newly 3 weeks spec	1022.	1018.03	{10., 2150.}	97.9188	0.812382	newly 3 weeks cumul	4122.	19 203.8	{2303., 32 260.}	383.363	0.535275	newly 5 weeks spec	367.	105.4	{0., 217.}	85.1989	1.52309	newly 5 weeks cumul	5105.	19 617.8	{2325., 32 301.}	305.957	0.490217	newly 47 weeks cumul	5925.	19 694.1	{2325., 32 302.}	256.76	0.454429
3	newly 3 weeks spec	703.	548.24	{0., 528.}	126.902	2.24774	newly 3 weeks cumul	2922.	9677.84	{386., 17 734.}	287.712	0.509156	newly 5 weeks spec	387.	199.33	{0., 84.}	131.832	2.47805	newly 5 weeks cumul	3733.	10 219.8	{386., 18 045.}	237.917	0.514625	newly 50 weeks cumul	4191.	10 422.6	{386., 18 045.}	218.032	0.523777
4	newly 3 weeks spec	301.	2038.68	{0., 5096.}	621.136	0.967408	newly 3 weeks cumul	2102.	12 530.9	{2465., 48 538.}	498.059	0.437445	newly 5 weeks spec	86.	549.96	{0., 1107.}	603.721	0.874593	newly 5 weeks cumul	2358.	14 196.4	{2488., 55 646.}	504.608	0.427808	newly 54 weeks cumul	2427.	14 675.6	{2491., 56 916.}	507.554	0.427632
5	newly 3 weeks spec	828.	567.09	{0., 1406.}	121.789	2.34493	newly 3 weeks cumul	3560.	8076.71	{497., 10 408.}	202.094	0.462256	newly 5 weeks spec	386.	203.13	{0., 222.}	123.101	2.53998	newly 5 weeks cumul	4530.	8609.35	{497., 15 268.}	177.899	0.517292	newly 50 weeks cumul	5288.	9018.3	{497., 20 033.}	166.738	0.559658
6	newly 3 weeks spec	764.	2167.5	{0., 3289.}	334.796	2.33661	newly 3 weeks cumul	3259.	6498.6	{647., 28 525.}	203.467	0.548471	newly 5 weeks spec	315.	1090.65	{0., 473.}	399.457	2.14928	newly 5 weeks cumul	4097.	9361.78	{674., 37 357.}	241.596	0.610933	newly 48 weeks cumul	4608.	10 483.5	{676., 37 598.}	244.504	0.636866
7	newly 3 weeks spec	276.	683.39	{56., 1002.}	197.17	0.43512	newly 3 weeks cumul	1666.	7652.9	{422., 8090.}	391.384	0.475047	newly 5 weeks spec	53.	233.65	{7., 253.}	413.415	0.619351	newly 5 weeks cumul	1818.	8253.06	{474., 8598.}	385.696	0.478825	newly 53 weeks cumul	1893.	8695.31	{482., 9168.}	391.061	0.48531
8	newly 3 weeks spec	397.	3980.95	{70., 15 279.}	966.108	0.933972	newly 3 weeks cumul	2114.	26 061.5	{2008., 81 555.}	1147.59	0.863675	newly 5 weeks spec	163.	511.9	{1., 1169.}	304.613	1.14029	newly 5 weeks cumul	2526.	28 041.2	{2042., 90 223.}	1026.17	0.827694	newly 47 weeks cumul	2724.	28 361.4	{2042., 91 210.}	956.604	0.801514
9	newly 3 weeks spec	565.	1099.36	{106., 1194.}	169.795	0.388717	newly 3 weeks cumul	2870.	6348.27	{816., 5144.}	176.044	0.309836	newly 5 weeks spec	200.	369.31	{26., 344.}	171.455	0.488179	newly 5 weeks cumul	3444.	7349.89	{844., 6961.}	173.5	0.325647	newly 52 weeks cumul	3765.	7882.59	{959., 9386.}	171.977	0.335842
10	newly 3 weeks spec	792.	1644.19	{6., 3075.}	186.789	0.827885	newly 3 weeks cumul	3481.	24 056.9	{628., 92 556.}	613.556	0.63819	newly 5 weeks spec	358.	198.82	{0., 335.}	99.2458	1.2922	newly 5 weeks cumul	4318.	24 813.8	{628., 93 615.}	498.032	0.586513	newly 46 weeks cumul	4964.	25 005.6	{628., 93 624.}	427.356	0.549512
11	newly 3 weeks spec	702.	469.46	{0., 654.}	106.265	2.03155	newly 3 weeks cumul	3890.	10 229.6	{1055., 10 962.}	204.327	0.323842	newly 5 weeks spec	225.	73.34	{0., 96.}	102.622	2.09438	newly 5 weeks cumul	4534.	10 474.9	{1070., 12 207.}	181.643	0.337662	newly 50 weeks cumul	4756.	10 543.3	{1070., 13 486.}	175.305	0.343417
12	newly 3 weeks spec	1213.	1402.71	{69., 4401.}	95.2638	0.5785	newly 3 weeks cumul	4897.	23 891.6	{6417., 72 885.}	393.499	0.474411	newly 5 weeks spec	613.	161.41	{1., 394.}	84.5922	1.19415	newly 5 weeks cumul	6341.	24 484.5	{6615., 73 301.}	292.385	0.381933	newly 46 weeks cumul	8505.	24 846.5	{6752., 73 318.}	201.311	0.286519
13	newly 3 weeks spec	527.	2533.16	{112., 7645.}	446.44	0.564343	newly 3 weeks cumul	2892.	7398.55	{491., 30 748.}	217.908	0.511142	newly 5 weeks spec	244.	1912.13	{31., 10 973.}	762.365	0.679903	newly 5 weeks cumul	3457.	11 765.6	{606., 43 833.}	300.704	0.530844	newly 51 weeks cumul	3655.	16 454.8	{744., 87 433.}	402.489	0.576483
14	newly 3 weeks spec	361.	977.99	{69., 3150.}	229.765	0.492388	newly 3 weeks cumul	1836.	11 206.4	{418., 27 168.}	551.825	0.57212	newly 5 weeks spec	115.	197.26	{10., 430.}	164.243	0.593181	newly 5 weeks cumul	2169.	11 796.7	{487., 28 485.}	485.49	0.552183	newly 53 weeks cumul	2408.	12 284.1	{516., 33 944.}	452.421	0.542318
15	newly 3 weeks spec	612.	986.59	{0., 986.}	207.449	2.1456	newly 3 weeks cumul	3189.	11 515.2	{1378., 30 109.}	299.57	0.359877	newly 5 weeks spec	228.	287.9	{0., 190.}	187.904	2.26482	newly 5 weeks cumul	3718.	12 372.6	{1378., 54 801.}	282.749	0.386165	newly 50 weeks cumul	4085.	12 580.3	{1378., 58 284.}	264.949	0.400266
16	newly 3 weeks spec	1144.	1218.32	{67., 2458.}	104.844	0.603689	newly 3 weeks cumul	4245.	26 833.5	{7344., 88 067.}	536.241	0.60975	newly 5 weeks spec	573.	172.84	{0., 296.}	102.29	1.75904	newly 5 weeks cumul	5611.	27 423.7	{7349., 88 508.}	393.871	0.507981	newly 46 weeks cumul	6762.	27 568.9	{7595., 88 519.}	313.545	0.436518
17	newly 3 weeks spec	602.	1363.8	{38., 873.}	200.827	0.488406	newly 3 weeks cumul	3587.	7036.93	{498., 3979.}	162.496	0.372034	newly 5 weeks spec	237.	469.61	{8., 481.}	191.152	0.680077	newly 5 weeks cumul	4153.	8314.33	{511., 5072.}	167.569	0.384538	newly 52 weeks cumul	4431.	8995.15	{511., 6778.}	170.257	0.393506
18	newly 3 weeks spec	80.	968.26	{17., 1178.}	1147.93	0.828379	newly 3 weeks cumul	978.	8310.56	{434., 8486.}	775.204	0.672151	newly 5 weeks spec	21.	232.8	{0., 580.}	1072.76	1.04608	newly 5 weeks cumul	1011.	8996.33	{434., 9009.}	815.016	0.692906	newly 56 weeks cumul	1018.	9427.03	{434., 9102.}	851.266	0.715058
19	newly 3 weeks spec	761.	2456.63	{51., 7427.}	291.184	0.653033	newly 3 weeks cumul	3302.	29 207.1	{2566., 94 626.}	795.733	0.637594	newly 5 weeks spec	360.	176.82	{0., 516.}	82.7778	1.21817	newly 5 weeks cumul	4108.	30 022.2	{2575., 96 784.}	647.612	0.594934	newly 47 weeks cumul	4733.	30 110.9	{2575., 96 872.}	557.173	0.566282
20	newly 3 weeks spec	252.	1263.8	{37., 2401.}	461.524	0.59986	newly 3 weeks cumul	2135.	12 325.3	{929., 13 391.}	508.139	0.509658	newly 5 weeks spec	37.	290.68	{1., 740.}	766.486	0.918573	newly 5 weeks cumul	2324.	13 191.1	{1002., 15 407.}	499.641	0.51339	newly 56 weeks cumul	2336.	13 639.4	{1002., 17 517.}	515.688	0.524445
21	newly 3 weeks spec	232.	769.06	{16., 1235.}	314.526	0.724213	newly 3 weeks cumul	1801.	9848.92	{490., 7890.}	483.956	0.611649	newly 5 weeks spec	64.	160.62	{0., 404.}	272.219	1.36915	newly 5 weeks cumul	1977.	10 350.5	{501., 8122.}	461.887	0.609386	newly 56 weeks cumul	2067.	10 557.	{501., 8552.}	449.804	0.609927
22	newly 3 weeks spec	1379.	1424.26	{0., 3705.}	115.796	1.1241	newly 3 weeks cumul	5215.	21 498.1	{2020., 95 366.}	336.779	0.428861	newly 5 weeks spec	709.	158.01	{0., 707.}	82.9887	1.82446	newly 5 weeks cumul	6926.	22 102.3	{2089., 95 387.}	255.307	0.405013	newly 46 weeks cumul	9186.	22 215.4	{2094., 95 387.}	190.995	0.394006
23	newly 3 weeks spec	467.	2204.5	{181., 4923.}	434.458	0.415785	newly 3 weeks cumul	3051.	5239.52	{853., 23 433.}	154.818	0.416698	newly 5 weeks spec	143.	1699.28	{35., 8975.}	1127.94	0.467805	newly 5 weeks cumul	3445.	9196.8	{1087., 42 756.}	242.542	0.416986	newly 51 weeks cumul	3590.	12 799.8	{1342., 87 583.}	322.972	0.417343
24	newly 3 weeks spec	99.	1356.1	{0., 2633.}	1338.28	1.15787	newly 3 weeks cumul	1045.	17 041.1	{723., 79 872.}	1546.63	0.813579	newly 5 weeks spec	29.	721.78	{0., 1081.}	2518.83	1.73198	newly 5 weeks cumul	1106.	18 641.3	{723., 80 647.}	1602.15	0.843942	newly 55 weeks cumul	1133.	23 021.8	{723., 86 822.}	1948.93	0.898143
25	newly 3 weeks spec	1111.	1369.48	{0., 1698.}	120.45	0.990587	newly 3 weeks cumul	4640.	21 877.6	{7404., 59 421.}	378.854	0.539429	newly 5 weeks spec	633.	452.24	{0., 323.}	139.659	1.88711	newly 5 weeks cumul	6102.	23 077.7	{7410., 60 083.}	286.032	0.446493	newly 46 weeks cumul	7554.	23 651.	{7410., 62 303.}	221.885	0.369928
26	newly 3 weeks spec	720.	2774.13	{190., 7070.}	316.399	0.578419	newly 3 weeks cumul	3694.	12 802.7	{741., 24 594.}	275.813	0.482367	newly 5 weeks spec	332.	1543.07	{67., 3570.}	402.593	0.700626	newly 5 weeks cumul	4491.	16 385.5	{1222., 33 975.}	291.901	0.515386	newly 48 weeks cumul	5251.	21 625.1	{2115., 44 002.}	333.549	0.57692
27	newly 3 weeks spec	1036.	771.11	{1., 1354.}	73.4508	0.908916	newly 3 weeks cumul	4214.	21 282.6	{7316., 62 858.}	407.826	0.532088	newly 5 weeks spec	467.	109.62	{0., 187.}	95.4818	1.8906	newly 5 weeks cumul	5302.	21 639.7	{7384., 63 024.}	311.172	0.44697	newly 46 weeks cumul	6355.	21 775.5	{7384., 63 025.}	246.094	0.37644
28	newly 3 weeks spec	1038.	3420.	{28., 13 969.}	300.557	0.699614	newly 3 weeks cumul	4164.	23 385.6	{2941., 82 408.}	477.294	0.524133	newly 5 weeks spec	683.	410.76	{0., 952.}	91.1303	1.33933	newly 5 weeks cumul	5692.	24 931.4	{2944., 87 673.}	360.873	0.473548	newly 47 weeks cumul	8123.	25 222.9	{2944., 87 990.}	244.984	0.419218
29	newly 3 weeks spec	584.	1000.77	{0., 597.}	215.529	1.46873	newly 3 weeks cumul	2514.	4365.79	{515., 3047.}	168.916	0.475867	newly 5 weeks spec	328.	498.52	{0., 223.}	211.848	2.20843	newly 5 weeks cumul	3268.	5578.94	{517., 3537.}	179.315	0.555547	newly 49 weeks cumul	4524.	6437.85	{517., 3694.}	168.673	0.667079
30	newly 3 weeks spec	466.	1479.47	{8., 768.}	307.187	0.735699	newly 3 weeks cumul	2784.	7973.19	{399., 29 463.}	261.621	0.487686	newly 5 weeks spec	120.	1018.27	{0., 288.}	830.342	0.862773	newly 5 weeks cumul	3116.	10 296.9	{400., 60 305.}	302.373	0.486897	newly 51 weeks cumul	3221.	12 243.7	{400., 64 769.}	349.059	0.486123
31	newly 3 weeks spec	874.	2187.37	{54., 3802.}	210.161	0.602235	newly 3 weeks cumul	3903.	11 147.4	{228., 17 391.}	239.018	0.584673	newly 5 weeks spec	482.	847.72	{23., 2248.}	143.531	0.612479	newly 5 weeks cumul	4981.	13 331.6	{285., 20 670.}	221.792	0.579759	newly 48 weeks cumul	5893.	15 030.	{344., 22 540.}	206.128	0.554018
32	newly 3 weeks spec	1191.	1813.58	{37., 3833.}	136.882	0.760373	newly 3 weeks cumul	4640.	24 950.8	{5239., 87 720.}	447.379	0.51416	newly 5 weeks spec	844.	290.25	{0., 692.}	97.5983	1.47916	newly 5 weeks cumul	6529.	25 955.8	{5254., 88 977.}	311.014	0.417045	newly 46 weeks cumul	9247.	26 174.1	{5254., 88 990.}	216.042	0.388789
33	newly 3 weeks spec	62.	1088.2	{14., 1025.}	1683.35	0.93324	newly 3 weeks cumul	424.	7668.71	{242., 8041.}	1725.82	0.933587	newly 5 weeks spec	6.	277.63	{0., 543.}	4560.5	1.23587	newly 5 weeks cumul	438.	8474.22	{243., 8454.}	1851.38	0.960666	newly 56 weeks cumul	438.	9198.14	{243., 8634.}	2016.32	0.987307
34	newly 3 weeks spec	175.	1036.86	{123., 2077.}	506.606	0.490803	newly 3 weeks cumul	1326.	6120.83	{749., 11 353.}	377.215	0.406923	newly 5 weeks spec	25.	371.49	{20., 729.}	1400.76	0.803027	newly 5 weeks cumul	1434.	7106.04	{840., 12 621.}	409.439	0.428167	newly 52 weeks cumul	1519.	7893.69	{867., 18 109.}	432.785	0.443026
35	newly 3 weeks spec	0.1	351.53	{0., 196.}	351 522.	1.63107	newly 3 weeks cumul	47.	7260.56	{861., 7048.}	15 350.7	1.77945	newly 5 weeks spec	0.1	325.32	{0., 36.}	325 334.	1.02694	newly 5 weeks cumul	47.	7916.45	{867., 11 522.}	16 746.2	1.80164	newly 56 weeks cumul	47.	9857.84	{867., 12 434.}	20 876.9	1.82533
36	newly 3 weeks spec	307.	1389.72	{16., 2347.}	410.866	0.670126	newly 3 weeks cumul	1782.	10 731.6	{273., 10 630.}	540.633	0.649777	newly 5 weeks spec	117.	358.7	{0., 961.}	300.034	1.00028	newly 5 weeks cumul	2037.	11 771.5	{288., 14 568.}	517.227	0.649256	newly 56 weeks cumul	2462.	12 358.3	{288., 19 467.}	443.563	0.62865
37	newly 3 weeks spec	611.	368.77	{2., 498.}	115.003	1.31502	newly 3 weeks cumul	3063.	2766.86	{469., 3602.}	91.5024	0.446204	newly 5 weeks spec	219.	103.39	{0., 117.}	115.685	2.25374	newly 5 weeks cumul	3644.	3060.85	{477., 3932.}	94.3911	0.500835	newly 49 weeks cumul	4006.	3214.66	{477., 4046.}	95.68	0.533756
38	newly 3 weeks spec	40.	2141.15	{125., 6697.}	5253.63	1.14497	newly 3 weeks cumul	901.	11 204.1	{3820., 25 416.}	1145.67	0.859196	newly 5 weeks spec	6.	839.84	{5., 2687.}	13 906.7	1.15445	newly 5 weeks cumul	919.	13 374.6	{4251., 32 732.}	1357.42	0.895508	newly 56 weeks cumul	919.	14 848.5	{4293., 36 022.}	1517.78	0.920958
39	newly 3 weeks spec	244.	667.15	{47., 1364.}	235.947	0.501506	newly 3 weeks cumul	1717.	8147.78	{388., 13 678.}	421.863	0.518732	newly 5 weeks spec	134.	130.91	{5., 293.}	107.261	0.759197	newly 5 weeks cumul	1991.	8551.79	{412., 14 595.}	377.743	0.508709	newly 53 weeks cumul	2147.	8718.27	{417., 14 722.}	355.062	0.505327
40	newly 3 weeks spec	154.	1633.76	{0., 3181.}	1048.26	1.17682	newly 3 weeks cumul	1047.	17 136.9	{716., 75 939.}	1550.07	0.835884	newly 5 weeks spec	17.	696.07	{0., 771.}	4115.94	1.74787	newly 5 weeks cumul	1131.	18 763.1	{716., 80 346.}	1572.94	0.853405	newly 55 weeks cumul	1136.	21 952.3	{716., 85 234.}	1846.41	0.908545
41	newly 3 weeks spec	226.	615.95	{24., 1276.}	249.562	0.609021	newly 3 weeks cumul	1612.	7350.54	{611., 8424.}	381.624	0.499273	newly 5 weeks spec	99.	164.69	{0., 321.}	189.04	1.26891	newly 5 weeks cumul	1842.	7811.84	{675., 8753.}	353.395	0.491633	newly 56 weeks cumul	1901.	8193.26	{679., 10 023.}	360.977	0.497303
42	newly 3 weeks spec	285.	994.73	{37., 2001.}	295.842	0.490905	newly 3 weeks cumul	1860.	8973.89	{203., 16 966.}	423.883	0.548946	newly 5 weeks spec	90.	319.15	{7., 409.}	334.322	0.674498	newly 5 weeks cumul	2101.	9809.48	{251., 18 096.}	407.475	0.543111	newly 53 weeks cumul	2198.	10 559.5	{304., 18 186.}	420.298	0.547599
43	newly 3 weeks spec	344.	1197.25	{23., 2738.}	320.677	0.634748	newly 3 weeks cumul	2328.	11 508.2	{339., 10 723.}	431.897	0.55999	newly 5 weeks spec	97.	313.13	{1., 704.}	327.804	0.997961	newly 5 weeks cumul	2595.	12 390.5	{352., 14 542.}	415.989	0.555537	newly 56 weeks cumul	2707.	13 005.6	{401., 19 473.}	418.984	0.557671
44	newly 3 weeks spec	177.	327.83	{0., 860.}	187.949	1.25414	newly 3 weeks cumul	1333.	9152.66	{1344., 10 489.}	595.188	0.680688	newly 5 weeks spec	67.	141.29	{0., 397.}	237.836	1.79502	newly 5 weeks cumul	1521.	9494.51	{1651., 11 507.}	531.351	0.653848	newly 55 weeks cumul	1548.	10 081.2	{2272., 18 083.}	556.534	0.675671
45	newly 3 weeks spec	708.	1591.79	{148., 496.}	235.99	0.505569	newly 3 weeks cumul	3626.	3726.57	{855., 2203.}	115.04	0.460614	newly 5 weeks spec	308.	1114.2	{25., 358.}	369.429	0.589336	newly 5 weeks cumul	4438.	6400.32	{1115., 2681.}	156.309	0.475733	newly 51 weeks cumul	4856.	8441.52	{1372., 5738.}	176.423	0.44629
46	newly 3 weeks spec	683.	1835.27	{127., 2631.}	239.589	0.426348	newly 3 weeks cumul	3807.	9462.52	{625., 24 414.}	198.341	0.347725	newly 5 weeks spec	255.	575.56	{21., 528.}	218.612	0.573765	newly 5 weeks cumul	4509.	11 071.1	{735., 25 755.}	199.874	0.359702	newly 52 weeks cumul	4663.	11 821.4	{827., 26 006.}	206.83	0.362666
47	newly 3 weeks spec	364.	1146.36	{191., 1161.}	245.137	0.253748	newly 3 weeks cumul	2257.	6684.32	{1899., 5629.}	210.588	0.228487	newly 5 weeks spec	75.	335.99	{24., 336.}	384.28	0.40448	newly 5 weeks cumul	2514.	7645.85	{2196., 7366.}	216.529	0.220641	newly 52 weeks cumul	2640.	8080.35	{2323., 9959.}	217.748	0.217147
48	newly 3 weeks spec	674.	2502.28	{34., 6788.}	339.187	0.798708	newly 3 weeks cumul	3437.	30 714.	{1666., 97 141.}	810.565	0.741813	newly 5 weeks spec	214.	238.24	{0., 677.}	111.888	1.17082	newly 5 weeks cumul	4062.	31 667.8	{1696., 99 249.}	697.776	0.706948	newly 47 weeks cumul	4384.	31 836.8	{1698., 99 333.}	644.81	0.6868
49	newly 3 weeks spec	230.	711.76	{29., 1262.}	251.722	0.518617	newly 3 weeks cumul	1489.	9390.05	{242., 13 459.}	562.181	0.577978	newly 5 weeks spec	69.	142.44	{3., 232.}	179.536	0.655894	newly 5 weeks cumul	1667.	9821.63	{253., 14 193.}	520.393	0.573534	newly 53 weeks cumul	1729.	10 013.7	{255., 14 266.}	510.201	0.578321
50	newly 3 weeks spec	221.	2072.69	{0., 5362.}	877.914	1.03004	newly 3 weeks cumul	1420.	11 740.9	{2340., 28 363.}	732.057	0.593085	newly 5 weeks spec	59.	760.99	{0., 1226.}	1244.66	0.939567	newly 5 weeks cumul	1550.	13 757.2	{2413., 32 674.}	792.692	0.599172	newly 54 weeks cumul	1593.	14 635.9	{2433., 33 750.}	823.839	0.60074
